# A case series of pediatric COVID-19 with complicated symptoms in Iran

**DOI:** 10.2217/fvl-2021-0091

**Published:** 2021-10-08

**Authors:** Fatemeh Cheraghali, Leila Barati, Dayan Amanian, Lobat Shahkar, Maryam Najafinejad, Hamed Naziri, Somayeh Shahabi, Alijan Tabarraei, Alireza Tahamtan

**Affiliations:** 1 ^1^Department of Pediatrics, School of Medicine, Taleghani Children’s Hospital, Golestan University of Medical Sciences, Gorgan, Iran; 2 ^2^Department of Radiology, School of Medicine, Golestan University of Medical Sciences, Gorgan, Iran; 3 ^3^Department of Microbiology, School of Medicine, Guilan University of Medical Sciences, Rasht, Iran; 4 ^4^Infectious Diseases Research Centre, Golestan University of Medical Sciences, Gorgan, Iran; 5 ^5^Department of Microbiology, School of Medicine, Golestan University of Medical Sciences, Gorgan, Iran

**Keywords:** case series, COVID-19, Iran, pediatrics, SARS-CoV-2

## Abstract

People in different age groups are susceptible to SARS-CoV-2 infection as a newly emerging virus. However, the clinical course, symptoms and disease outcome vary from case to case. Although COVID-19 is usually milder in children than adults, some studies reported nonspecific symptoms. Here, we report eight pediatric cases of COVID-19 admitted in the Taleghani Children Hospital in Gorgan city, north of Iran, with complicated symptoms. The current case series poses several challenges to the pediatricians regarding the pediatric cases of COVID-19. As most literature relating to adults are not always transferable to children, clinicians should be warned about such presentations among children with COVID-19.

Today, COVID-19 has spread worldwide, caused by SARS-CoV-2. While all people in different age groups are susceptible to the newly emerging virus infection, the clinical course and disease outcome vary from case to case [1–3]. Due to weakened immune responses, older patients with underlying diseases are more affected by COVID-19 and associated with much higher case-fatality rates [4,5]. Preliminary evidence suggests that children are less prone to severe disease and death, possibly because of their immune system status with relatively low levels of inflammatory cytokines and having more pulmonary stem cells that can repair the damaged cells [6–8]. Despite this, there is not enough data to determine the exact behavior of SARS-CoV-2 infection in pediatrics. Although the disease is usually milder in children than in adults, some studies reported nonspecific symptoms. As the COVID-19 situation appears to be changing, clinicians should be warned of pediatrics infection. Here, we report eight pediatric cases of COVID-19 admitted in the Taleghani Children Hospital in Gorgan city, north of Iran, with complicated symptoms.

## Cases description

### Case 1

**Figure 1.**
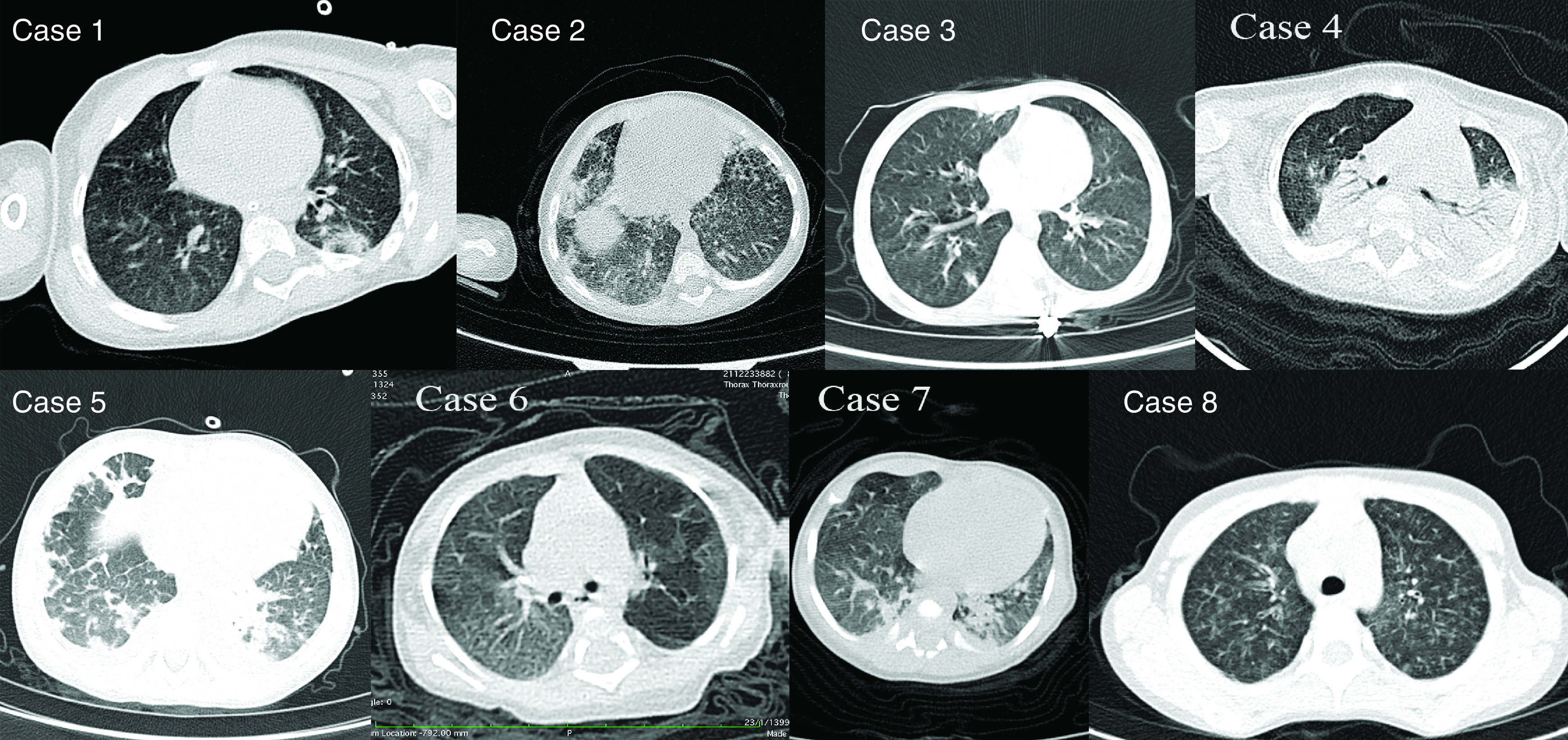
Computed tomography scan obtained from cases. Case 1: Patchy consolidation in the superior segment of the left-lower lobe and mild mosaic attenuation in both lungs. Case 2: Peripherally located patchy consolidations and ground-glass opacities in the basal segments of right-lower lobe (RLL) and inferior aspects of right-middle lobe and lingula. Case 3: Peripherally located patchy consolidation in the RLL and centrilobular ground-glass opacities in both lungs. Case 4: Collapse-consolidations in the posterior aspects of both lungs and patchy ground-glass opacity in the right-upper lobe. Case 5: Peripherally located patchy consolidations along with interlobular septal thickening. Case 6: Ground-glass opacities created regions of differing pulmonary attenuation (mosaic attenuation pattern) along with mild peribronchial wall thickening. Case 7: Collapse consolidations in posteromedial aspects of both lower lobes and mosaic attenuation in lungs. Case 8: Diffuse centrilobular ground-glass opacities and mild peribronchial wall thickening in both lungs.

A 34-month-old male was admitted with a history of fever, recurrent seizure and low-level consciousness. Fever began 4 days before the seizure. Clinical and laboratory findings of all cases are shown in Table 1. In lumbar puncture (LP), the cerebrospinal fluid (CSF) appeared clear with normal protein and glucose level, normal white and red blood cell count (WBC and RBC), and negative for bacterial growth. The brain MRI proposed viral encephalitis with possible parenchymal hemorrhagic components. The molecular tests for HSV-1 and 2 were negative. Although CSF analysis did not reveal any evidence of CNS infection, SARS-CoV-2 RNA was detected in the nasopharyngeal swab and CSF with the same cycle threshold. After that, patchy consolidation in the superior segment of the left-lower lobe was observed in the spiral chest computed-tomography (CT) scan (Figure 1). At admission, treatment was started with dexamethasone and wide-spectrum antibiotics, but after SARS-CoV-2 detection, it changed to hydroxychloroquine, azithromycin, intravenous immunoglobulin (IVIG), lopinavir, ritonavir and caltra. His situation has not improved, and he got cerebral palsy. More details of this case have been published in our earlier work as a case report article [9].

**Table 1.** Clinical and laboratory findings of all cases at admission.

	Case 1	Case 2	Case 3	Case 4	Case 5	Case 6	Case 7	Case 8
Age (months)	34	12	33	8	54	7	2	72
Gender	Male	Male	Male	Male	Male	Female	Male	Male

**Clinical findings**								
Main symptoms	Seizures, loss of consciousness	Fever, seizure, abdominal protrusion	Respiratory distress, fever	Respiratory distress, cyanosis	Fever, severe weakness, lethargy, anorexia, stomach ache, edema	Respiratory distress, fever	Respiratory distress, and no weight gain	Respiratory distress, fever
Underlying diseases	–	FTT	ALL, FTT	Immunodeficiency FTT	–	Severe FTT	Congenital heart disease	Asthma
Outcome	Severe neurologic morbidity	Complete improvement	Death	Death	Complete improvement	Death	Partial improvement	Complete improvement

**Laboratory findings**								
WBC (cells/μl)	23,300	30,000	31,300	11,800	17,700	7700	12,100	10,400
RBC (mil/mm^3^)	5.41	3.56	3.66	2.79	3.87	4.39	3.37	4.51
Hb (g/dl)	12.3	7.1	8.9	7.7	10.6	12.4	9.8	12.6
Hct (%)	36.7	22.7	27.8	22.8	31.7	36.1	29.9	35.9
MCV (fl)	67.84	63.76	73.16	81.72	81.91	82.23	88.72	79.6
MCH (pg)	22.74	19.94	23.42	27.6	27.39	28.25	29.08	27.9
MCHC (g/dl)	23.51	31.28	32.01	33.77	33.44	34.35	32.78	35.1
PLT	727,000	173,000	107,000	76,000	29,000	540,000	454,000	812,000
PMN	67%	22%	25%	68%	90%	43%	35%	55%
Lymph	20%	74%	75%	32%	10%	57%	65%	40%
BUN (mg/dl)	7	7	12	12	17	13	12	11
Creatinine (mg/dl)	0.5	0.5	0.6	0.6	0.6	0.6	0.6	0.6
Na (mg/dl)	142	138	140	140	140	138	137	139
K (mg/dl)	4.8	5	4.5	4.9	4	4.2	6.5	4.4
CRP	+1	+1	1+	+3	+1	Negative	Negative	3+
ESR (mm/h)	35	85	17	10	18	6	7	101
LDH	2826	651	1503	1366	651	867	712	483
AST	154	171	49	55	36	31	27	21
ALT	111	65	20	26	35	52	22	8

**CSF analysis**								
Glucose	65	55						
Protein	39	39						
WBC	2	95						
Poly		10%						
Lymph		90%						
Culture	Negative	Negative						

**RT-PCR result**								
1st test	Positive	Negative	Negative	Positive	Positive	Negative	Negative	Positive
2nd test		Negative	Suspicious			Positive	Positive	
3rd test		Positive	Positive					

ALL: Acute lymphoblastic leukemia; ALT: Alanine transaminase; AST: Aspartate transaminase; BUN: Blood urea nitrogen; CRP: C-reactive protein; CSF: Cerebrospinal fluid; ESR: Erythrocyte sedimentation rate; FTT: Failure to thrive; Hb: Hemoglobin; Hct: Hematocrit; LDH: Lactate dehydrogenase; MCH: Mean corpuscular hemoglobin; MCHC: Mean corpuscular hemoglobin concentration; MCV: Mean corpuscular volume; PLT: Platelet; PMN: Polymorphonuclear leukocytes; RBC: Red blood cell; RT-PCR: Real-time reverse transcription PCR; WBC: White blood cell.

### Case 2

A 12-month-old male was referred because of fever, seizure and anemia while suffering from fever and diarrhea for 3 days. The seizure was upward gaze with generalized tonic–clonic movement in a few seconds. In the initial examination, abdominal distention and hepatosplenomegaly were observed. At admission, laboratory findings showed leukocytosis with lymphocytosis and decreased hemoglobin. The main findings of LP were pleocytosis with 90% lymph and negative for bacterial growth. The molecular test for SARS-CoV-2 and HSV-1 and 2 in CSF was negative. With suspicion of meningoencephalitis, wide-spectrum antibiotics were used. CT scan showed peripherally located patchy consolidations and ground-glass opacities in the basal segments of the right-lower lobe and inferior aspects of the right-middle lobe and lingula (Figure 1). SARS-CoV-2 and TB tests in respiratory samples were negative. Because of the reverse in CD4/CD8 ratio, an HIV test was ordered and reported as negative. The molecular test for SARS-CoV-2 again was negative. Abdominal sonography and CT scan showed hepatosplenomegaly and lymphadenopathy in the *para*-aorta and celiac trunk. Although his brain CT scan and MRI findings were normal, bone marrow aspiration revealed mild erythroid hyperplasia. Nasopharyngeal swab became positive for SARS-CoV-2 on day 18. Treatment was started with hydroxychloroquine, and the patient became well and was discharged.

### Case 3

A 33-month-old male was admitted due to unilateral massive pleural effusion, respiratory distress and fever. Both a microbial examination in pleural fluid and the SARS-CoV-2 test in nasopharyngeal swab were negative. The main laboratory finding was leukocytosis with polymorphonuclear dominance. Pleural biopsy and bone marrow aspiration revealed acute lymphoblastic leukemia T cells. The patient was referred to the oncology department, and chemotherapy was performed for 3 weeks. One week later with sustained fever, lethargy and cough, the SARS-CoV-2 test was done, and the result was suspicious. His CT scan revealed peripherally located patchy consolidation in the right-lower lobe and centrilobular ground-glass opacities in both lungs (Figure 1). Treatment was started with cotrimoxazole, meropenem, vancomycin, nystatin, hydroxychloroquine, dexamethasone, pantoprazole and albumin. Four days later, the SARS-CoV-2 test became positive. Before receiving the RT-PCR result, the patient was transferred to the pediatric intensive care unit (PICU) because of respiratory distress and decreased O_2_ saturation, and he expired due to severe illness and anemia.

### Case 4

An 8-month-old male was referred to the pediatric emergency department with fever, productive cough, tachypnea, vomiting and diarrhea. At admission, bilateral whizzing and respiratory distress were heard with a stethoscope. He was born preterm and had normal delivery and development. The patient had a history of 33 days of hospitalization at birth and two-times due to pneumonia at 5 and 7 months. On arrival, the child had a respiratory rate of 61 per minute, a heart rate of 150 and O_2_ saturation of 75%. The level of total IgM and IgG was lower, which indicated humoral immune deficiency. Collapse consolidations in the posterior aspects of both lungs and patchy ground-glass opacity in the right-upper lobe were observed in the CT scan (Figure 1). TB and influenza tests were negative. The patient was transferred to the PICU, and treatment started with meropenem, vancomycin and cotrimoxazole. Nasopharyngeal swab became positive for SARS-CoV-2 RNA on day 4. He received IVIG and linezolid during the hospitalization, and hydroxychloroquine and ciprofloxacin were also added to the treatment. On day 20, the patient had a bilateral pneumothorax, and a chest tube was implanted, but the patient expired 2 days later.

### Case 5

A 4.5-year-old male was hospitalized because of fever, severe weakness, lethargy, anorexia and abdominal pain. In the initial examination, the patient had a little edematous and tachypnea. At admission, the main laboratory findings were severe thrombocytopenia, anemia with hemoglobin of 7, leukocytosis with 90% polymorphonuclear and albumin of 2.2. His lung CT scan revealed peripherally located patchy consolidations along with interlobular septal thickening (Figure 1). With suspicion of COVID-19, the physician ordered the SARS-CoV-2 RT-PCR test. Due to the patient’s condition, albumin and caltra drugs were prescribed. Later day, the nasopharyngeal swab became positive for SARS-CoV-2 RNA. With the start of treatment, his condition improved, and he was discharged 6 days later in good condition.

### Case 6

A 7-month-old female was admitted due to whizzing, fever and food intolerance. She weighed 1.5 kg at birth and 3 kg at the time of admission, but the development was normal. In previous hospitalizations, there was no found reason for her failure to thrive. Initial physical examination showed a little tachypnea with normal O_2_ saturation. At admission, laboratory findings were normal. In CT scan, ground-glass opacities created regions of differing pulmonary attenuation (mosaic attenuation pattern) along with mild peribronchial wall thickening were observed (Figure 1). Thus, COVID-19 was proposed by the attended physician, but the primary SARS-CoV-2 RT-PCR test was negative. Cefotaxime was used for the first 3 days of hospitalization, and the respiratory condition relatively improved. On the fourth day of hospitalization, she suddenly developed apnea, was transferred to the PICU and was intubated. Treatment was started with hydroxychloroquine, IVIG, meropenem and vancomycin. Later day, the nasopharyngeal swab became positive for SARS-CoV-2 RNA. Unfortunately, the patient expired 3 days later.

### Case 7

A 2-month-old male was hospitalized with symptoms such as tachypnea, heart murmur, crying, lethargy, poor feeding and no weight gain. His mother reported that she had been wheezing and restless for about a month. The main finding of initial laboratory findings was anemia. The patient chest x-ray proposed bronchopneumonia, so antibiotic therapy started. There was no COVID-19 history in his family, and his primary COVID-19 test was negative. The CT scan showed subsegmental collapse consolidation in posteromedial aspects of both lower lobes and mosaic attenuation in lungs (Figure 1). According to the CT report, the COVID-19 test was repeated, and the nasopharyngeal swab became positive for SARS-CoV-2 RNA. After 10 days of antibiotic therapy, the patient was discharged in relatively good condition.

### Case 8

A 6-year-old male was referred with symptoms and signs such as fever, cough and respiratory distress that started about 10 days prior. The patient was asthmatic and under treatment with salbutamol spray and corticosteroids. He had a history of hospitalization due to pneumonia last year. His body temperature reached over 40°C, O_2_ saturation was under 91%, and he had respiratory distress even while resting. The patient had no history of travel or contact with COVID-19 cases. The main laboratory findings were thrombocytosis and erythrocyte sedimentation rate of 101. The CT scan showed diffuse centrilobular ground-glass opacities and mild peribronchial wall thickening in both lungs (Figure 1). The patient was reported to be positive in the SARS-CoV-2 RT-PCR test. Treatment started with ceftriaxone, hydroxychloroquine and oxygen therapy. Finally, he was discharged in relatively good condition.

## Discussion

The notifiable COVID-19 cases mainly were among adults, and pediatric patients were rarely reported. Importantly, preliminary clinical findings showed that children usually presented with mild or asymptomatic infections [10]. In theory, children are also susceptible to SARS-CoV-2 infection, and lower likelihood to get severe disease and death may be related to the children staying at home during the pandemic and having less contact with the source of infection. Other factors may include their still-developing immune system and having more pulmonary stem cells that can repair the injured cells. While the COVID-19 situation appears to be changing in pediatrics, its clinical profile in this population is less known [11]. This is a case series report on COVID-19 in children from Iran. These cases were in 2 months to 7 years, indicating no age group is safe from infection with SARS-CoV-2. Although with a small number of cases, we found that male children are more affected than females. This may be because of anatomical, genetic, hormonal and immunological differences. Notably, the expression level of ACE2 is found to be more in males than females [8].

The most important finding to come from the present analysis is the complicated pediatrics symptoms for COVID-19. Of the eight cases, five had respiratory symptoms, two had fever, seizures, low-level consciousness and one had edema, abdominal pain and severe weakness. Cases 1 and 2 were admitted with neurological manifestations, and this was different in that cases. While CSF analysis of case 1 was normal, the virus was detected in CSF. While the main LP findings in case 2 were pleocytosis with 90% lymph, CSF was negative for SARS-CoV-2. Neurological manifestations have been suggested as a presenting symptom or complication of COVID-19 [12], but only a few studies detected SARS-CoV-2 in the CSF [9,13]. Our case 1 represents the presence of SARS-CoV-2 in both the respiratory tract and CSF and highlights the importance of consideration of neurological complications as unknown signs of COVID-19. More studies are needed to guide the pediatricians further and confirm the CNS infection of SARS-CoV-2, neurologic manifestations and how the virus enters the CNS.

In these cases, inconsistent with the Liu *et al.*’s study [14], severe leukocytosis was observed in four patients, two of whom recovered and two died. The former two had polymorphonuclear, while the latter two had lymph dominance. No lymphopenia or leukopenia was seen that is inconsistent with other adult studies, indicating a different manifestation of the COVID-19 in pediatrics. The RT-PCR test was reported positive at admission only in four positive and in two cases were positive in the third test. Although the RT-PCR test confirmed all subjects as the ‘criterion-referenced’ for COVID-19 detection [15], we cannot rule out the potential of nosocomial infection or false-negative results, especially in cases that the test was negative for the first or second time. It is hard to determine common clinical characteristics in children with COVID-19, and it is unclear whether there is a common biomarker due to the small number of cases. Although the overall symptoms are relatively mild in children, the CT scan profiles are similar to adults.’

## Conclusion

The current case series poses several challenges to the pediatricians regarding the pediatrics COVID-19. As most literature relating to adults are not always transferable to children, clinicians should be warned for such presentations among pediatrics with COVID-19. It is recommended that children with unusual clinical symptoms be screened for SARS-CoV-2 infection. Further research and reports are crucial to help us understand the clinical characteristics and natural history of COVID-19 in children.

**Summary points**

- As an emerging virus, all people in different age groups are susceptible to SARS-CoV-2 infection.
- Although COVID-19 is usually milder in children than in adults, some studies reported nonspecific symptoms.
- The current case series poses several challenges to the pediatricians regarding the pediatrics COVID-19.
- Of the eight cases, five had respiratory symptoms, two had a fever, seizures and low-level consciousness, and one had edema, abdominal pain and severe weakness.
- Cases 1 and 2 highlight the importance of consideration of neurological complications as unknown signs of COVID-19.
- As most literature relating to adults are not always transferable to children, clinicians should be warned for such presentations among pediatrics with COVID-19.

## References

[1] Kay FU, Abbara S. The many faces of COVID-19: spectrum of imaging manifestations. Radiol: Cardiothorac. Imaging. 2(1), e200037 (2020). 33779634 10.1148/ryct.2020200037 PMC7233435

[2] Xu X-W, Wu X-X, Jiang X-G Clinical findings in a group of patients infected with the 2019 novel coronavirus (SARS-CoV-2) outside of Wuhan, China: retrospective case series. BMJ 368, m606 (2020). 32075786 10.1136/bmj.m606 PMC7224340

[3] Teymoori-Rad M, Samadizadeh S, Tabarraei A Ten challenging questions about SARS-CoV-2 and COVID-19. Expert Rev. Respir. Med. 14(9), 881–888 (2020). 32536226 10.1080/17476348.2020.1782197

[4] Chen N, Zhou M, Dong X Epidemiological and clinical characteristics of 99 cases of 2019 novel coronavirus pneumonia in Wuhan, China: a descriptive study. Lancet 395(10223), 507–513 (2020). 32007143 10.1016/S0140-6736(20)30211-7 PMC7135076

[5] Wang D, Hu B, Hu C Clinical characteristics of 138 hospitalized patients with 2019 novel coronavirus-infected pneumonia in Wuhan, China. JAMA 323(11), 1061–1069 (2020). 32031570 10.1001/jama.2020.1585 PMC7042881

[6] Sinha IP, Harwood R, Semple MG COVID-19 infection in children. Lancet. Res. Med. 8(5), 446–447 (2020). 10.1016/S2213-2600(20)30152-1 PMC7154504 32224304

[7] Kelvin AA, Halperin S. COVID-19 in children: the link in the transmission chain. Lancet. Infect. Dis. 20(6), 633–634 (2020). 32220651 10.1016/S1473-3099(20)30236-X PMC7156154

[8] Samadizadeh S, Masoudi M, Rastegar M COVID-19: why does disease severity vary among individuals? Respir. Med. 180, 106356 (2021). 33713961 10.1016/j.rmed.2021.106356 PMC7934673

[9] Cheraghali F, Tahamtan A, Hosseini SA Case Report: detection of SARS-CoV-2 from cerebrospinal fluid in a 34-month-old child with encephalitis. Front. Pediatric. 9, 194 (2021). 10.3389/fped.2021.565778 PMC8093448 33959568

[10] Zheng F, Liao C, Fan Q-H Clinical characteristics of children with coronavirus disease 2019 in Hubei, China. Curr. Med. Sci. 40(2), 275–280 (2020). 32207032 10.1007/s11596-020-2172-6 PMC7095065

[11] Tahamtan A, Tavakoli-Yaraki M, Salimi V. Opioids/cannabinoids as a potential therapeutic approach in COVID-19 patients. Expert Rev. Respir. Med. 14(10), 965–967 (2020). 32576053 10.1080/17476348.2020.1787836 PMC7441794

[12] Filatov A, Sharma P, Hindi F Neurological complications of coronavirus disease (COVID-19): encephalopathy. Cureus 12(3), e7352 (2020). 32328364 10.7759/cureus.7352 PMC7170017

[13] Moriguchi T, Harii N, Goto J A first case of meningitis/encephalitis associated with SARS-coronavirus-2. IJID 94, 55–58 (2020). 32251791 10.1016/j.ijid.2020.03.062 PMC7195378

[14] Liu W, Zhang Q, Chen J Detection of Covid-19 in children in early January 2020 in Wuhan, China. N. Engl. J. Med. 382(14), 1370–1371 (2020). 32163697 10.1056/NEJMc2003717 PMC7121643

[15] Tahamtan A, Ardebili A. Real-time RT-PCR in COVID-19 detection: issues affecting the results. Expert Rev. Mol. Diagn. 20(5), 453–454 (2020). 32297805 10.1080/14737159.2020.1757437 PMC7189409

